# Gene expression analysis in lymphoblasts derived from patients with autism spectrum disorder

**DOI:** 10.1186/2040-2392-2-9

**Published:** 2011-05-26

**Authors:** Yuka Yasuda, Ryota Hashimoto, Hidenaga Yamamori, Kazutaka Ohi, Motoyuki Fukumoto, Satomi Umeda-Yano, Ikuko Mohri, Akira Ito, Masako Taniike, Masatoshi Takeda

**Affiliations:** 1 Department of Psychiatry, Osaka University Graduate School of Medicine, D3, 2-2, Yamadaoka, Suita, 565-0871, Osaka, Japan; 2 CREST (Core Research for Evolutionary Science and Technology) of JST (Japan Science and Technology Agency), 4-1-8, Honcho, Kawaguchi, Saitama, 332-0112, Japan; 3 Molecular Research Center for Children's Mental Development, United Graduate School of Child Development, Osaka University, Kanazawa University and Hamamatsu University School of Medicine, D3, 2-2, Yamadaoka, Suita, Osaka, 565-0871, Japan; 4 Department of Molecular Neuropsychiatry, Osaka University Graduate School of Medicine, 2-2, Yamadaoka, Suita, Osaka, 565-0871, Japan; 5 Division of Developmental Neuroscience, United Graduate School of Child Development, Osaka University, Kanazawa University and Hamamatsu University School of Medicine, 2-2, Yamadaoka, Suita, Osaka, 565-0871, Japan

## Abstract

**Background:**

The autism spectrum disorders (ASDs) are complex neurodevelopmental disorders that result in severe and pervasive impairment in the development of reciprocal social interaction and verbal and nonverbal communication skills. In addition, individuals with ASD have stereotypical behavior, interests and activities. Rare mutations of some genes, such as neuroligin (*NLGN*) 3/4, neurexin (*NRXN*) 1, *SHANK3, MeCP2 *and *NHE9*, have been reported to be associated with ASD. In the present study, we investigated whether alterations in mRNA expression levels of these genes could be found in lymphoblastoid cell lines derived from patients with ASD.

**Methods:**

We measured mRNA expression levels of *NLGN3/4, NRXN1, SHANK3, MeCP2, NHE9 *and *AKT1 *in lymphoblastoid cells from 35 patients with ASD and 35 healthy controls, as well as from 45 patients with schizophrenia and 45 healthy controls, using real-time quantitative reverse transcriptase polymerase chain reaction assays.

**Results:**

The mRNA expression levels of *NLGN3 *and *SHANK3 *normalized by *β-actin *or *TBP *were significantly decreased in the individuals with ASD compared to controls, whereas no difference was found in the mRNA expression level of *MeCP2, NHE9 *or *AKT1*. However, normalized *NLGN3 *and *SHANK3 *gene expression levels were not altered in patients with schizophrenia, and expression levels of *NLGN4 *and *NRXN1 *mRNA were not quantitatively measurable in lymphoblastoid cells.

**Conclusions:**

Our results provide evidence that the *NLGN3 *and *SHANK3 *genes may be differentially expressed in lymphoblastoid cell lines from individuals with ASD compared to those from controls. These findings suggest the possibility that decreased mRNA expression levels of these genes might be involved in the pathophysiology of ASD in a substantial population of ASD patients.

## Background

Autism spectrum disorder (ASD), also known as pervasive developmental disorder (PDD), is defined as severe and pervasive impairments in the development of reciprocal social interaction and verbal and nonverbal communication skills. These disorders are also characterized by stereotypical behavior, interests and activities. The lifetime morbidity rate of ASD is 0.2% to 1.0% across studies [1]. In addition, twin and family studies of ASD have demonstrated a high heritability of approximately 90% [2], indicating that ASD is a heterogeneous condition that is likely to result from the combined effects of multiple genetic factors interacting with environmental factors. Recent genetic studies have identified several vulnerability loci and genetic mutations that cause ASD. One of the most striking revelations is the important role of genes that encode proteins at the neuronal synapse [3].

Rare mutations in the neuroligin 3 (*NLGN3*) and neuroligin 4 (*NLGN4*) genes, which map to chromosomes Xq13 and Xp22.3, have been reported in some patients with ASD and other neurodevelopmental impairments [4-8]. A particular mutation of *NLGN3 *(Arg451Cys) is known to cause a defect in protein processing of *NLGN3 *[9]. In addition, a particular mutation of *NLGN4 *(1186insT) causes a frameshift mutation that leads to premature termination of *NLGN4 *(D396X), resulting in a loss of 421 amino acids (51% of the protein) [4]. Neuroligins, which are postsynaptically localized cell adhesion molecules, play a crucial role in organizing excitatory glutamatergic and inhibitory GABAergic synapses in the mammalian brain by interacting with presynaptic β-neurexins (*NRXN*), thereby triggering the formation of functional presynaptic structures in contacting axons [10]. Mutations of the neurexin 1 (*NRXN1*) gene, at the chromosome locus 2q32, have been found in individuals with ASD [11-14]. Furthermore, *de novo *copy number variation analysis revealed deletion of the *NRXN1*-containing gene region in ASD [15]. The binding of *NRXN1 *and *NLGN *genes mediates synaptic development [16]. Interestingly, a mutation of *NLGN3 *results in a disruption of the ability to bind to *NRXN *[9]. In addition, neuroligins interact with a postsynaptic scaffolding protein, SHANK3, which is also implicated in ASD [17] and is located on the telomeric terminal of chromosome 22q13.3. Shank proteins couple neurotransmitter receptors, ion channels and other membrane proteins to the actin cytoskeleton and G protein-coupled signaling pathways, and they also play a role in synapse formation and dendritic spine maturation [18]. Deletion or translocation of the genomic locus, which includes the *SHANK3 *gene, and *de novo *mutations of the *SHANK3 *gene result in premature stop codons and have been found in ASD [17,19,20].

In a study of consanguineous autism families, Morrow *et al. *[21] observed a relationship between ASD and alterations in the sodium/hydrogen exchanger 9 (*NHE9*) gene. Specifically, they found a nonsense mutation in patients with ASD that is a heterozygous CGA-to-TGA transition, changing arginine 423 to a stop codon [21]. The *NHE9 *gene is located on chromosome 3q24 and is one of the families of Na^+^/H^+ ^exchangers that regulate ion flux across membranes [22]. Rett syndrome is another PDD, and the methyl-CpG-binding protein 2 (*MeCP2*) gene is a causal gene for Rett syndrome. *MeCP2 *is a transcriptional repressor that binds to methylated CpG dinucleotides generally located at gene promoters and recruits histone deacetylase 1 and other proteins involved in chromatin repression [23]. *De novo *mutations of the *MeCP2 *gene located on chromosome Xp28 occur in 80% of female patients with Rett syndrome [24]. Some evidence of dysregulation of the phosphatidylinositol 3-kinase (PI3K)/AKT pathway is implicated in ASD, despite the fact that no mutation which causes ASD has been reported in association with the *AKT1 *gene. The expression and phosphorylation and/or activation of AKT were found to be decreased in the autistic brain [25]. The *PTEN *gene (phosphatase and tensin homolog deleted on chromosome 10) is a major negative regulator of the PI3K/AKT pathway, and *PTEN *mutations have been linked to ASD [26].

Recently, several studies have suggested that lymphoblastoid cells can be used to detect biologically plausible correlations between candidate genes and neuropsychiatric diseases, including Rett syndrome [27], nonspecific X-linked mental retardation [28], bipolar disorder [29], fragile X syndrome [30,31] and dup(15q) [32]. In the present study, we compared mRNA expression levels of various genes in blood-derived lymphoblastoid cells from individuals with ASD and healthy controls.

## Methods

### Participants

We obtained mRNA samples from patients with ASD, patients with schizophrenia and healthy controls from the research bioresource of the Human Brain Phenotype Consortium in Japan (http://www.sp-web.sakura.ne.jp/consortium.html). The ASD cohort consisted of 35 patients with ASD and healthy controls (Table 1). Patients with ASD and patients with schizophrenia were recruited from both outpatient and inpatient services at Osaka University Hospital. Each ASD patient was diagnosed by at least two trained child psychiatrists and/or child neurologists according to the *Diagnostic and Statistical Manual of Mental Disorders, Fourth Edition-Text Revision *(DSM-IV-TR) criteria based on unstructured or semistructured behavioral observations of the patients and interviews with the patients and their parents or caregivers. During the interview, the Pervasive Developmental Disorders Autism Society Japan Rating Scale (PARS) [33] and the Japanese version of the Asperger's Questionnaire [34] were used to assist in the evaluation of ASD-specific behaviors and symptoms. PARS is a semistructured interview that is composed of 57 questions in eight domains of the characteristics of children with PDD, which was developed by the Autism Society Japan. The clinicians who diagnosed the individuals were trained in the use of PARS. Twenty individuals met the full criteria for autistic disorder, 11 met the criteria for Asperger syndrome and four for PDD-not otherwise specified (PDD-NOS). Among the patients with ASD, 11 had a low intelligence quotient (IQ) ( < 70). The schizophrenia cohort consisted of 45 patients with schizophrenia and 45 age- and sex-matched healthy controls (Table 2). Each patient with schizophrenia received a consensus diagnosis by at least two trained psychiatrists according to the DSM-IV-TR criteria using the structured clinical interview (SCID) for DSM-IV.

**Table 1:** Demographic information for the ASD and control cohorts^a^

Demographics	ASD (*n *= 35)	Controls (*n *= 35)	*P *value
Sex, M/F	27/8	26/9	χ^2 ^= 0.078 (1, *N *= 70), *P *= 0.78
Mean age, years ( ± SD)	12.9 (12.4)	34.8 (9.7)	*U *= 86, *P *= 0.60 × 10^-9^, *Z *= -6.19
Age range, years	3 to 63	21 to 65	
Number of ASD (with IQ < 70)	35 (11)	0	
Number of Autism (with IQ < 70)	20 (10)	-	
Number of Asperger's syndrome (with IQ < 70)	11 (0)	-	
Number with PDD-NOS (with IQ < 70)	4 (1)	-	

ASD: autism spectrum disorder, M: male, F: female, IQ: intelligence quotient; PDD-NOS: pervasive developmental disorder not otherwise specified. Data are means ± SD unless otherwise specified. Differences in clinical characteristics were analyzed using the χ^2 ^test for gender and the Mann-Whitney *U *test for age.

**Table 2:** Demographic information for schizophrenia and control cohorts^a^

Demographics	Schizophrenia (*n *= 45)	Controls (*n *= 45)	*P *value
Sex, M/F	26/19	26/19	χ^2 ^= 0 (1, *N *= 90), *P *= 1.0
Mean age, years ( ± SD)	37.9 (1.6)	38.1 (1.7)	*U *= 988.5, *P *= 0.9, *Z *= -0.2
Age range, years	21 to 65	21 to 65	
Estimated premorbid IQ (JART50)	100.8 (9.3)	105.4 (8.4)	*U *= 687, *P *= 0.009, *Z *= -2.6

M: male, F: female, IQ: intelligence quotient; JART50: Japanese Adult Reading Test: Japanese version of the National Adult Reading Test. Data are means ± SD unless otherwise specified. Differences in clinical characteristics were analyzed using the χ^2 ^test for gender and the Mann-Whitney *U *test for age.

A detailed description of healthy controls was given in previous reports [35,36]. Briefly, controls were biologically unrelated Japanese participants. Healthy controls were screened using the SCID for the *Diagnostic and Statistical Manual, Fourth Edition*, Axis I Disorders, Non-Patient version (SCID-I/NP) and were excluded if they (1) had neurological or medical conditions that could potentially affect the central nervous system, (2) had any psychiatric diseases and/or received psychiatric medication, (3) had first- or second-degree relatives with psychiatric disease or (4) presented with an IQ < 70. IQ data were collected using the Japanese version of the full-scale Wechsler Adult Intelligence Scale (WAIS)-III or the full-scale Wechsler Intelligence Scale for Children-Third Edition (WISC-III) [37,38].

Following description of the study, written informed consent was obtained from each individual (or, when appropriate, his/her guardians). This study was carried out in accordance with the World Medical Association's Declaration of Helsinki and was approved by the ethics committee at Osaka University.

### Immortalization of lymphocytes and RNA extraction

Isolation of lymphocytes from blood and lymphocyte immortalization using Epstein-Barr virus (EBV) were entrusted to SRL of Tokyo, Japan. Immortalized, patient-derived lymphocytes were grown in culture media supplemented with 20% fetal bovine serum. Total RNA was extracted from cell pellets using the RNeasy Mini Kit (Qiagen K.K., Tokyo, Japan). The total RNA yield was determined by absorbance at 260 nm, and RNA quality was analyzed using agarose gel electrophoresis.

### DNase treatment and reverse transcriptase reaction

Total RNA was treated with DNase to remove contaminating genomic DNA using DNase Treatment & Removal Reagents (Ambion, Austin, TX, USA) according to the manufacturer's protocol. Total RNA (10 μg) treated with DNase was used in a 50-μL reverse transcriptase reaction to synthesize cDNA with the SuperScript First-Strand Synthesis System for RT-PCR (Invitrogen, Carlsbad, CA, USA) according to the manufacturer's protocol. Briefly, total RNA (10 μg) was denatured with 1 mM deoxyribonucleotide triphosphate (dNTP) and 5 ng/μL random hexamers at 65°C for 5 minutes. After the addition of 10xRT buffer (20 mM Tris-HCl (pH 8.4) and 50 mM KCl final concentration; Invitrogen), MgCl_2 _(5 mM final concentration), dithiothreitol (10 mM final concentration), RNaseOUT Recombinant Ribonuclease Inhibitor (100 U; Invitrogen) and SuperScript III Reverse Transcriptase (125 U; Invitrogen), the reaction mixture was incubated at 25°C for 10 minutes, at 42°C for 40 minutes and at 70°C for 15 minutes. RNase H (5 U) was added to the reaction mixture and incubated at 37°C for 20 minutes to stop the reaction.

### Real-time quantitative RT-PCR

The Pre-Developed TaqMan Assay Reagent kit (Applied Biosystems, Foster City, CA, USA) was used to measure mRNA expression levels of *NLGN3, NLGN4, NRXN1, SHANK3, MeCP2, NHE9, AKT1 *and housekeeping genes (*β-actin *and *TBP*). Primers were purchased from Applied Biosystems (gene name: assay ID, transcript ID, target region; *NLGN3*: Hs01043809_m1, NM_181303.1, Exon4-5; *NLGN4*: Hs00535592_m1, NM_020742.2, Exon1-2; *NRXN1*: Hs00985123_m1, NM_001135659.1, Exon22-23; *SHANK3*: Hs01586468_m1, NM_001080420.1, Exon22-23; *MECP2*: Hs00172845_m1, NM_004992.3, Exon2-3; *NHE9*: Hs00543518_m1, NM_173653.3, Exon7-8; *AKT1*: Hs00920503_m1, NM_001014432.1, Exon13-14; β*-*actin: 4326315E, NM_001101, no region indicated; *TBP*: 4326322E, NM_003194, no region indicated). Expression levels of these genes were measured by real-time quantitative reverse transcriptase polymerase chain reaction (qRT-PCR) using an ABI Prism 7900 Sequence Detection System (Applied Biosystems) with a 384-well format as previously described [39,40]. Each 20-μL PCR reaction contained 6 μL of cDNA, 900 nM concentrations of each primer, a 250 nM concentration of probe and 10 μL of TaqMan Universal PCR Master Mix containing AmpliTaq Gold DNA Polymerase and AmpErase Uracil N-glycosylase (all from Applied Biosystems), as well as dNTP with deoxyuridine triphosphate, passive reference and optimized buffer components. The PCR cycling conditions were 50°C for 2 minutes, 95°C for 10 minutes, 40 cycles of 95°C for 15 seconds and 59°C or 60°C for 1 minute. PCR data were obtained by using Sequence Detector software (SDS version 2.1; Applied Biosystems) and quantified using a standard curve method. This software plotted the real-time fluorescence intensity and selected the threshold within the linear phase of the amplicon profile. The software plotted a standard curve of the cycle at threshold (*C*_t_) (where the fluorescence generated within a reaction crossed the threshold) versus the quantity of RNA. All samples were measured using a single plate per target gene, and their *C*_t _values were in the linear range of the standard curve. Sample quantities were predicted by *C*_t _values. Experiments were typically performed three times in triplicate, and each gene expression level was taken as the average of three independent experiments. The individual expression level of each target gene normalized by a housekeeping gene (raw target gene expression level divided by raw housekeeping gene expression level) was used for statistical analysis.

### Statistical analyses

Statistical analyses were carried out using SPSS for Windows version 16.0 software (SPSS Japan Inc., Tokyo, Japan). Group comparisons of demographic data were performed using the c^2 ^test for one categorical variable (sex) or the Mann-Whitney *U *test for continuous variables as appropriate. Differences in mRNA transcript levels between the groups were analyzed using the Mann-Whitney *U *test. The Bonferroni correction for multiple tests was applied to assess the mRNA transcript levels on the number of genes (five). All *P *values reported are based on two-tailed tests. Statistical significance was defined as *P *< 0.05.

## Results

**Figure 1:**
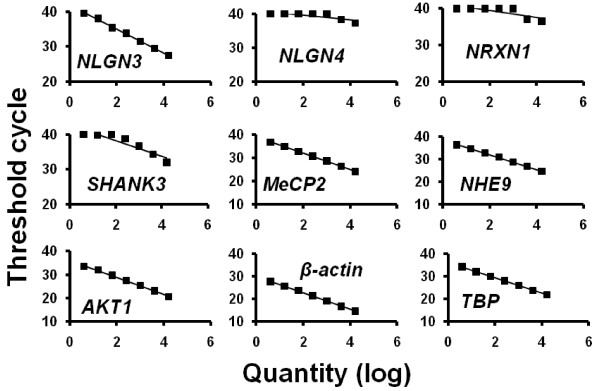
**Standard curves for target genes and housekeeping genes**. Standard curves for *NLGN3, NLGN4, NRXN1, SHANK3, MeCP2, NHE9, AKT1 *and two housekeeping genes (*β-actin *and *TBP*). The highest quantity represents an amount of cDNA prepared from 300 ng of total RNA in the polymerase chain reaction.

Standard curves for the seven target genes (*NLGN3, NLGN4, NRXN1, SHANK3, MeCP2, NHE9 *and *AKT1*) and the two housekeeping genes (*β-actin *and *TBP*) were prepared using serial dilutions (1:4) of pooled cDNA from 300 ng of total RNA derived from immortalized lymphoblasts (Figure 1). The *R*^2 ^values of the standard curves were more than 0.99 (*NLGN3, MeCP2, NHE9, AKT1, β-actin *and *TBP*), 0.87 (*SHANK3*), 0.64 (*NRXN1*) and 0.63 (*NLGN4*). Although the *SHANK3 *gene expression was relatively low, it was measurable in our sample. On the other hand, we did not further analyze *NLGN4 *and *NRXN1 *gene expression, as the expression levels of the two genes were too low to quantify using this method.

**Figure 2:**
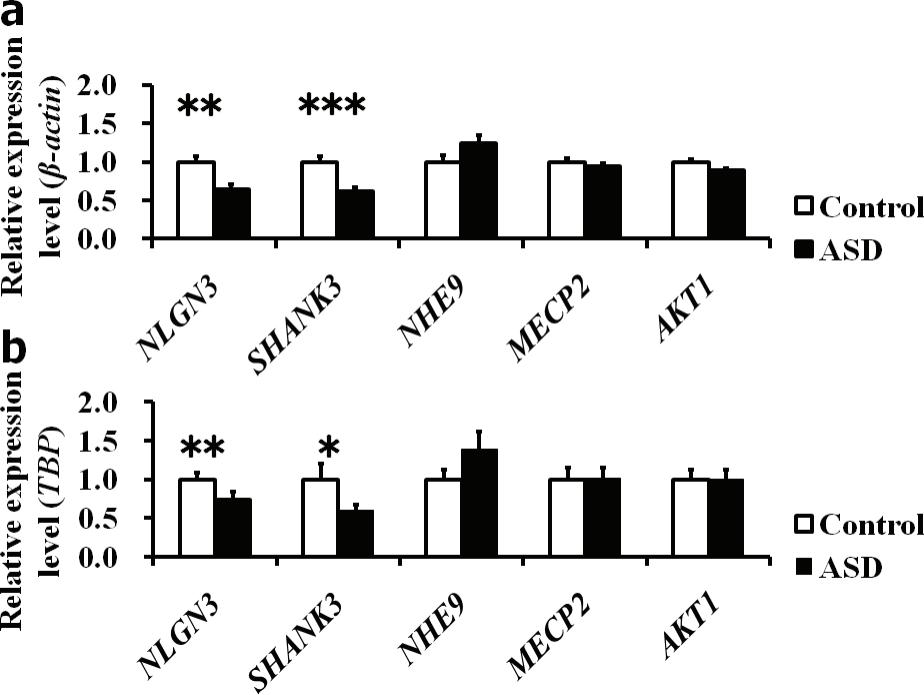
**Expression analysis of *NLGN3, SHANK3, NHE9, MeCP2 *and *AKT1 *in autism spectrum disorder**. Mean relative mRNA expression level scores normalized by housekeeping gene *β*-*actin ***(a) **or *TBP ***(b) **in the autism spectrum disorder (ASD) group and the control group are shown. Bars represent the standard error of the mean. Differences between the groups in expression levels of the five genes were analyzed by using the Mann-Whitney *U *test. *Post hoc *comparisons were performed by using the Bonferroni correction. **P < 0.01 and ***P < 0.001.

Using immortalized lymphoblastoid cells from 35 individuals with ASD and 35 controls, we quantified the mRNA expression levels of the *NLGN3, SHANK3, NHE9, MeCP2 *and *AKT1 *genes normalized by two housekeeping genes, *β-actin *and *TBP *(Figure 2). The mRNA expression levels of the *NLGN3 *gene normalized by *β-actin *or *TBP *were decreased by 35% or 26%, respectively, in individuals with ASD (*β-actin*: *P *= 0.00024; *TBP*: *P *= 0.00089). The mRNA expression levels of the *SHANK3 *gene normalized by *β-actin *or *TBP *were also decreased in individuals with ASD by 39% or 40%, respectively (*β-actin*: *P *= 0.000036; *TBP*: *P *= 0.0061). The mRNA expression levels of the *NHE9 *gene were increased by 24% (*P *= 0.052: normalized by *β-actin*) and 39% (*P *= 0.048: normalized by *TBP*). There was no significant difference in mRNA expression levels of the *MeCP2 *gene normalized by *β-actin *or *TBP *between the two groups (*P *> 0.1). The mRNA expression levels of the *AKT1 *gene were decreased by 11% (*P *= 0.03: normalized by *β-actin*); however, those levels were not altered when normalized by *TBP *(*P *= 0.45). After correction for multiple tests, mRNA expression levels of *NLGN3 *and *SHANK3 *remained significantly lower in individuals with ASD than in healthy controls (*NLGN3*: corrected *P *= 0.0012, normalized by *β-actin*, corrected *P *= 0.0045, normalized by *TBP*; *SHANK3*: corrected *P *= 0.00018, normalized by *β-actin*, corrected *P *= 0.03, normalized by *TBP*). However, the altered expression level of *NHE9 *or *AKT1 *was no longer significant after the correction for multiple tests (*P *> 0.1).

**Figure 3:**
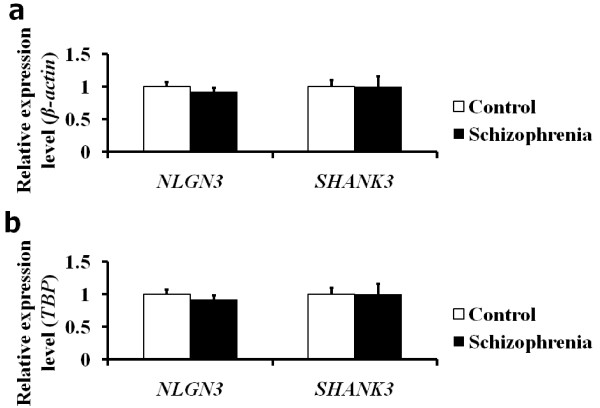
**Expression analysis of the *NLGN3 *and *SHANK3 *genes in patients with schizophrenia**. Mean relative mRNA expression level scores normalized by housekeeping gene *β-actin ***(a) **or *TBP ***(b) **in the schizophrenia group and the control group are shown. Bars represent standard error of the mean. Differences in expression levels of the two genes between the groups were analyzed by using the Mann-Whitney *U *test.

We next measured *NLGN3 *and *SHANK3 *mRNA expression levels in immortalized lymphoblastoid cells from 45 patients with schizophrenia and 45 healthy controls to examine the disease specificity of the differential expression levels between patients and healthy controls (Figure 3). We found that the mRNA expression levels for these two genes normalized by *β-actin *or *TBP *were not significantly different between patients with schizophrenia and healthy controls (*P *> 0.2). These results suggest that reduced levels of *NLGN3 *and *SHANK3 *mRNA expression might be associated with ASD but not with schizophrenia.

## Discussion

In this study, we found that the mRNA expression levels of *NLGN3 *and *SHANK3 *were significantly lower in individuals with ASD than in healthy controls. Mutations of causal genes are rare, and they have been found to be associated with specific types of ASD. Our findings suggest that not only rare mutations of the causal genes but also functional alterations in the transcriptional activity of these genes might be associated with the pathophysiology of ASD. The *NLGN3 *and *SHANK3 *genes are synapse-related genes and were found to be affected in ASD, whereas other genes, including *NHE9 *and *MeCP2*, do not play major roles at the synapse and were not found to be affected in ASD. These findings suggest that impairments in synaptic function might be associated with the pathophysiology of ASD.

Reduced expression of the *NLGN3 *and *SHANK3 *genes in lymphoblasts of individuals with ASD is consistent with previous reports indicating that mutations of these genes cause reduced expression or loss of function of the protein. Since the *NLGN3 *gene is located in chromosome X, there may be expressional difference between genders. However, no significant difference of *NLGN3 *gene expression normalized by *β-actin *or *TBP *was observed with regard to gender in healthy controls or individuals with ASD (*P *> 0.05). This might be due to inactivation of one X chromosome in females [41]. There are several possibilities that might explain the reduced expression of the *NLGN3 *and *SHANK3 *genes in ASD. First, our sporadic ASD cases might have mutations, polymorphisms or copy number variations in the *NLGN3 *or *SHANK3 *genes, which could result in reduced expression of these genes. Second, mutations or polymorphisms in genes that regulate the expression of *NLGN3 *or *SHANK3 *might contribute to the observed reduction in expression of the *NLGN3 *or *SHANK3 *genes. To our knowledge, although the regulation of *NLGN3 *by other genes has not been reported, there are some reports in the literature describing the regulation of *SHANK3 *gene expression. For example, *SHANK3 *expression is regulated by DNA methylation [42,43]. In addition, *SHANK3 *is one of the predicted targets of dysregulated microRNA (miRNA), and altered miRNA expression levels were found in postmortem brain from autism patients [44]. Further epigenetic analyses might elucidate the mechanisms of reduced *SHANK3 *expression.

Some findings of gene expression in lymphoblastoid cell lines are in conflict with those of previous studies. For example, Beri *et al. *[42] reported that *SHANK3 *is not expressed in EBV-transformed human lymphoblastoid cell lines in an investigation of tissue-specific *SHANK3 *gene expression and DNA methylation. By using lymphoblastoid cells from autism patients, Talebizadeh *et al. *[8] detected novel splice isoforms of *NLGN4*. There are methodological differences between previous studies and our study. *SHANK3 *gene expression in the previous study [42] was analyzed by using a conventional RT-PCR method; however, we measured the expression levels of *SHANK3 *gene by using a real-time qRT-PCR method (the TaqMan method). Furthermore, the expression level of *SHANK3 *was relatively low, which is shown in the standard curve in Figure 1. It is possible that the sensitivity of our real-time qRT-PCR method to detect the *SHANK3 *gene expression level might be higher than that of a conventional RT-PCR method. On the other hand, we could not quantitatively measure the *NLGN4 *and *NRXN1 *genes by using the real-time qRT-PCR method. However, there were slight expressions of these genes in lymphoblastoid cell lines when we used a large quantity of cDNA for the real-time qRT-PCR (Figure 1). Unfortunately, the small expression levels of these genes made it impossible to quantitatively measure the gene expressions in our sample. This may explain possible discrepancies of the gene expression findings of previous studies and our results.

There are several limitations of this study. First, our positive results might have arisen from sample bias due to non-age-matched samples, although the Japanese are a relatively homogeneous population, so the use of non-age-matched samples is unlikely to explain our findings. Second, our sample size might not be small for type I errors but small for type II errors. There is a possibility of type II errors in mRNA expression differences of *NHE9, MECP2 *and *AKT1 *between individuals with ASD and healthy controls and expression differences of *NLGN3 *and *SHANK3 *between individuals with schizophrenia and healthy controls. In particular, *NHE9 *might be increased in individuals with ASD, as the expression level of *NHE9 *was marginally significant before correction for multiple testing. Thus, replication studies using a larger sample size are needed before a firm conclusion can be drawn. Third, we did not perform a mutation search for the examined genes in our sample to replicate the association between the examined genes and ASD and how the causal or risk variants of the genes regulate the gene expression. As the previous evidence for candidate genes of ASD are based on rare mutations and/or copy number variations of the genes, it might be difficult to find a mutation in our 35 individuals with ASD for analysis of the variant effects on the gene expression in this study. A mutation search study of these candidate genes should be done in future studies. Fourth, the IQ scores in the ASD group were lower than those in the healthy control group, so reduced gene expression could be related to lower IQ. However, lower expression of the *NLGN3 *or *SHANK3 *genes was not found in individuals with schizophrenia who had lower premorbid IQ scores, and no expression difference was observed in individuals with ASD and mental retardation versus individuals with ASD but without mental retardation (data not shown). Taken together, the reduced gene expression in ASD might be specific to ASD, although other neuropsychiatric diseases, such as attention-deficit/hyperactivity disorder, mental retardation, major depression and bipolar disorder, should be examined in future studies. The ASD cases in this study were consistent with idiopathic autism diagnosed on the basis of clinical features. We did not include individuals with Rett syndrome and the other syndromic autisms, such as multiple sclerosis, which could explain why we did not find altered expression of *MeCP2 *in this cohort. Our results suggest that the *MeCP2 *gene may not be associated with the common pathology of ASD, while *NLGN3 *and *SHANK3 *may be. Because lymphoblastoid cell lines are not neuronal cells, some of our findings might not reflect the pathophysiology in ASD brains. Further studies investigating these limitations are warranted.

## Conclusions

Our study reveals reduced levels of *NLGN3 *and *SHANK3 *mRNA expression in lymphoblastoid cell lines derived from individuals with ASD, but not from those of individuals with schizophrenia. These results are consistent with findings that rare mutations of these genes in specific cases cause loss of function, suggesting that reduction of *NLGN3 *and *SHANK3 *mRNA expression could be related to the pathophysiology of ASD in a substantial population of patients. Although there are several limitations present in this study, lymphoblastoid cell lines may still allow investigation of the pathophysiology of ASD. Further analyses are required, such as a mutation analysis of the *NLGN3 *and *SHANK3 *genes and the genes regulating their expression, in addition to studies designed to elucidate the mechanisms of this reduced expression.

## Abbreviations

ASD, autism spectrum disorder; DSM-IV-TR, *Diagnostic and Statistical Manual of Mental Disorders, Fourth Edition*-*Text Revision*; F, female; IQ, intelligence quotient; JART50, Japanese Adult Reading Test; M, male; *MeCP2*, methyl-CpG-binding protein 2; *NHE9*, sodium/hydrogen exchanger 9; *NLGN*, neuroligin; *NRXN*, neurexin; PARS, Pervasive Developmental Disorders Autism Society Japan Rating Scale; PDD, pervasive developmental disorder; PDD-NOS, pervasive developmental disorder not otherwise specified; SCID, structured clinical interview; SCID-I/NP, *Diagnostic and Statistical Manual, Fourth Edition*, Axis I Disorders, Non-Patient version; SD, standard deviation; WAIS-III, Wechsler Adult Intelligence Scale-III; WISC-III, Wechsler Intelligence Scale for Children-Third Edition.

## Competing interests

The authors declare that they have no competing interests.

## Authors' contributions

RH supervised the entire project; collected the data; wrote the manuscript; was critically involved in the design, analysis and interpretation of the data; and was responsible for performing the literature review. YY was critically involved in the collection and analysis of the data, contributed to the editing of the final manuscript and contributed intellectually to the interpretation of the data. HY, SU and AI were involved in the mRNA measurements and collection of the majority of the data. KO, MF, IM, MTan and MTak were heavily involved in the collection of the majority of the data and contributed intellectually to the interpretation of the data. All authors reviewed the manuscript before submission and approved the final manuscript.

## References

[B1] Levy SE Mandell DS Schultz RT Autism Lancet 2009 374 1627 1638 10.1016/S0140-6736(09)61376-3 19819542 PMC2863325

[B2] Lichtenstein P Carlström E Råstam M Gillberg C Anckarsäter H The genetics of autism spectrum disorders and related neuropsychiatric disorders in childhood Am J Psychiatry 2010 167 1357 1363 10.1176/appi.ajp.2010.10020223 20686188

[B3] Walsh CA Morrow EM Rubenstein JL Autism and brain development Cell 2008 135 396 400 10.1016/j.cell.2008.10.015 18984148 PMC2701104

[B4] Jamain S Quach H Betancur C Råstam M Colineaux C Gillberg IC Soderstrom H Giros B Leboyer M Gillberg C Bourgeron T Paris Autism Research International Sibpair Study: Mutations of the X-linked genes encoding neuroligins NLGN3 and NLGN4 are associated with autism Nat Genet 2003 34 27 29 10.1038/ng1136 12669065 PMC1925054

[B5] Laumonnier F Bonnet-Brilhault F Gomot M Blanc R David A Moizard MP Raynaud M Ronce N Lemonnier E Calvas P Laudier B Chelly J Fryns JP Ropers HH Hamel BC Andres C Barthélémy C Moraine C Briault S X-linked mental retardation and autism are associated with a mutation in the *NLGN4 *gene, a member of the neuroligin family Am J Hum Genet 2004 74 552 557 10.1086/382137 14963808 PMC1182268

[B6] Lawson-Yuen A Saldivar JS Sommer S Picker J Familial deletion within NLGN4 associated with autism and Tourette syndrome Eur J Hum Genet 2008 16 614 618 10.1038/sj.ejhg.5202006 18231125

[B7] Yan J Oliveira G Coutinho A Yang C Feng J Katz C Sram J Bockholt A Jones IR Craddock N Cook EH Jr Vicente A Sommer SS Analysis of the neuroligin 3 and 4 genes in autism and other neuropsychiatric patients Mol Psychiatry 2005 10 329 332 10.1038/sj.mp.4001629 15622415

[B8] Talebizadeh Z Lam DY Theodoro MF Bittel DC Lushington GH Butler MG Novel splice isoforms for NLGN3 and NLGN4 with possible implications in autism J Med Genet 2006 43 e21 16648374 10.1136/jmg.2005.036897 PMC2564526

[B9] Comoletti D De Jaco A Jennings LL Flynn RE Gaietta G Tsigelny I Ellisman MH Taylor P The Arg451Cys-neuroligin-3 mutation associated with autism reveals a defect in protein processing J Neurosci 2004 24 4889 4893 10.1523/JNEUROSCI.0468-04.2004 15152050 PMC6729460

[B10] Craig AM Kang Y Neurexin-neuroligin signaling in synapse development Curr Opin Neurobiol 2007 17 43 52 10.1016/j.conb.2007.01.011 17275284 PMC2820508

[B11] Feng J Schroer R Yan J Song W Yang C Bockholt A Cook EH Jr Skinner C Schwartz CE Sommer SS High frequency of neurexin 1β signal peptide structural variants in patients with autism Neurosci Lett 2006 409 10 13 10.1016/j.neulet.2006.08.017 17034946

[B12] Kim HG Kishikawa S Higgins AW Seong IS Donovan DJ Shen Y Lally E Weiss LA Najm J Kutsche K Descartes M Holt L Braddock S Troxell R Kaplan L Volkmar F Klin A Tsatsanis K Harris DJ Noens I Pauls DL Daly MJ MacDonald ME Morton CC Quade BJ Gusella JF Disruption of neurexin 1 associated with autism spectrum disorder Am J Hum Genet 2008 82 199 207 10.1016/j.ajhg.2007.09.011 18179900 PMC2253961

[B13] Autism Genome Project Consortium Szatmari P Paterson AD Zwaigenbaum L Roberts W Brian J Liu XQ Vincent JB Skaug JL Thompson AP Senman L Feuk L Qian C Bryson SE Jones MB Marshall CR Scherer SW Vieland VJ Bartlett C Mangin LV Goedken R Segre A Pericak-Vance MA Cuccaro ML Gilbert JR Wright HH Abramson RK Betancur C Bourgeron T Gillberg C Mapping autism risk loci using genetic linkage and chromosomal rearrangements Nat Genet 2007 39 319 328 10.1038/ng1985 17322880 PMC4867008

[B14] Yan J Noltner K Feng J Li W Schroer R Skinner C Zeng W Schwartz CE Sommer SS Neurexin 1α structural variants associated with autism Neurosci Lett 2008 438 368 370 10.1016/j.neulet.2008.04.074 18490107

[B15] Glessner JT Wang K Cai G Korvatska O Kim CE Wood S Zhang H Estes A Brune CW Bradfield JP Imielinski M Frackelton EC Reichert J Crawford EL Munson J Sleiman PM Chiavacci R Annaiah K Thomas K Hou C Glaberson W Flory J Otieno F Garris M Soorya L Klei L Piven J Meyer KJ Anagnostou E Sakurai T Autism genome-wide copy number variation reveals ubiquitin and neuronal genes Nature 2009 459 569 573 10.1038/nature07953 19404257 PMC2925224

[B16] Dean C Dresbach T Neuroligins and neurexins: linking cell adhesion, synapse formation and cognitive function Trends Neurosci 2006 29 21 29 10.1016/j.tins.2005.11.003 16337696

[B17] Durand CM Betancur C Boeckers TM Bockmann J Chaste P Fauchereau F Nygren G Rastam M Gillberg IC Anckarsäter H Sponheim E Goubran-Botros H Delorme R Chabane N Mouren-Simeoni MC de Mas P Bieth E Rogé B Héron D Burglen L Gillberg C Leboyer M Bourgeron T Mutations in the gene encoding the synaptic scaffolding protein SHANK3 are associated with autism spectrum disorders Nat Genet 2007 39 25 27 10.1038/ng1933 17173049 PMC2082049

[B18] Vessey JP Karra D More than just synaptic building blocks: scaffolding proteins of the post-synaptic density regulate dendritic patterning J Neurochem 2007 102 324 332 10.1111/j.1471-4159.2007.04662.x 17596209

[B19] Bonaglia MC Giorda R Borgatti R Felisari G Gagliardi C Selicorni A Zuffardi O Disruption of the ProSAP2 gene in a t(12;22)(q24.1;q13.3) is associated with the 22q13.3 deletion syndrome Am J Hum Genet 2001 69 261 268 10.1086/321293 11431708 PMC1235301

[B20] Moessner R Marshall CR Sutcliffe JS Skaug J Pinto D Vincent J Zwaigenbaum L Fernandez B Roberts W Szatmari P Scherer SW Contribution of *SHANK3 *mutations to autism spectrum disorder Am J Hum Genet 2007 81 1289 1297 10.1086/522590 17999366 PMC2276348

[B21] Morrow EM Yoo SY Flavell SW Kim TK Lin Y Hill RS Mukaddes NM Balkhy S Gascon G Hashmi A Al-Saad S Ware J Joseph RM Greenblatt R Gleason D Ertelt JA Apse KA Bodell A Partlow JN Barry B Yao H Markianos K Ferland RJ Greenberg ME Walsh CA Identifying autism loci and genes by tracing recent shared ancestry Science 2008 321 218 223 10.1126/science.1157657 18621663 PMC2586171

[B22] Nakamura N Tanaka S Teko Y Mitsui K Kanazawa H Four Na^+^/H^+ ^exchanger isoforms are distributed to Golgi and post-Golgi compartments and are involved in organelle pH regulation J Biol Chem 2005 280 1561 1572 15522866 10.1074/jbc.M410041200

[B23] Chahrour M Zoghbi HY The story of Rett syndrome: from clinic to neurobiology Neuron 2007 56 422 437 10.1016/j.neuron.2007.10.001 17988628

[B24] Renieri A Meloni I Longo I Ariani F Mari F Pescucci C Cambi F Rett syndrome: the complex nature of a monogenic disease J Mol Med 2003 81 346 354 12750821 10.1007/s00109-003-0444-9

[B25] Sheikh AM Malik M Wen G Chauhan A Chauhan V Gong CX Liu F Brown WT Li X BDNF-Akt-Bcl2 antiapoptotic signaling pathway is compromised in the brain of autistic subjects J Neurosci Res 88 2641 2647 10.1002/jnr.22416 20648653

[B26] Zhou J Blundell J Ogawa S Kwon CH Zhang W Sinton C Powell CM Parada LF Pharmacological inhibition of mTORC1 suppresses anatomical, cellular, and behavioral abnormalities in neural-specific *Pten *knock-out mice J Neurosci 2009 29 1773 1783 10.1523/JNEUROSCI.5685-08.2009 19211884 PMC3904448

[B27] Horike S Cai S Miyano M Cheng JF Kohwi-Shigematsu T Loss of silent-chromatin looping and impaired imprinting of *DLX5 *in Rett syndrome Nat Genet 2005 37 31 40 10.1038/ng1570 15608638

[B28] Meloni I Muscettola M Raynaud M Longo I Bruttini M Moizard MP Gomot M Chelly J des Portes V Fryns JP Ropers HH Magi B Bellan C Volpi N Yntema HG Lewis SE Schaffer JE Renieri A *FACL4*, encoding fatty acid-CoA ligase 4, is mutated in nonspecific X-linked mental retardation Nat Genet 2002 30 436 440 10.1038/ng857 11889465

[B29] Iwamoto K Kakiuchi C Bundo M Ikeda K Kato T Molecular characterization of bipolar disorder by comparing gene expression profiles of postmortem brains of major mental disorders Mol Psychiatry 2004 9 406 416 10.1038/sj.mp.4001437 14743183

[B30] Brown V Jin P Ceman S Darnell JC O'Donnell WT Tenenbaum SA Jin X Feng Y Wilkinson KD Keene JD Darnell RB Warren ST Microarray identification of FMRP-associated brain mRNAs and altered mRNA translational profiles in fragile X syndrome Cell 2001 107 477 487 10.1016/S0092-8674(01)00568-2 11719188

[B31] Nishimura Y Martin CL Vazquez-Lopez A Spence SJ Alvarez-Retuerto AI Sigman M Steindler C Pellegrini S Schanen NC Warren ST Geschwind DH Genome-wide expression profiling of lymphoblastoid cell lines distinguishes different forms of autism and reveals shared pathways Hum Mol Genet 2007 16 1682 1698 10.1093/hmg/ddm116 17519220

[B32] Baron CA Tepper CG Liu SY Davis RR Wang NJ Schanen NC Gregg JP Genomic and functional profiling of duplicated chromosome 15 cell lines reveal regulatory alterations in UBE3A-associated ubiquitin-proteasome pathway processes Hum Mol Genet 2006 15 853 869 10.1093/hmg/ddl004 16446308

[B33] Yamada A Suzuki M Kato M Tanaka S Shindo T Taketani K Akechi T Furukawa TA Emotional distress and its correlates among parents of children with pervasive developmental disorders Psychiatry Clin Neurosci 2007 61 651 657 10.1111/j.1440-1819.2007.01736.x 18081627

[B34] Wakabayashi A Tojo Y Baron-Cohen S Wheelwright S [The Autism-Spectrum Quotient (AQ) Japanese version: evidence from high-functioning clinical group and normal adults] [in Japanese] Shinrigaku Kenkyu 2004 75 78 84 15724518 10.4992/jjpsy.75.78

[B35] Yasuda Y Hashimoto R Ohi K Fukumoto M Takamura H Iike N Yoshida T Hayashi N Takahashi H Yamamori H Morihara T Tagami S Okochi M Tanaka T Kudo T Kamino K Ishii R Iwase M Kazui H Takeda M Association study of *KIBRA *gene with memory performance in a Japanese population World J Biol Psychiatry 2010 11 852 857 10.3109/15622971003797258 20509760

[B36] Hashimoto R Ohi K Yasuda Y Fukumoto M Iwase M Iike N Azechi M Ikezawa K Takaya M Takahashi H Yamamori H Okochi T Tanimukai H Tagami S Morihara T Okochi M Tanaka T Kudo T Kazui H Iwata N Takeda M The impact of a genome-wide supported psychosis variant in the *ZNF804A *gene on memory function in schizophrenia Am J Med Genet B Neuropsychiatr Genet 2010 153B 1459 1464 10.1002/ajmg.b.31123 20957649

[B37] Committee JW-IP Japanese Wechsler Intelligence Scale for Children 1998 Tokyo: Nihon Bunka Kagakusha

[B38] Wechsler D Wechsler Intelligence Scale for Children-Third Edition Manual 1991 New York: Psychological Corp

[B39] Hashimoto R Straub RE Weickert CS Hyde TM Kleinman JE Weinberger DR Expression analysis of neuregulin-1 in the dorsolateral prefrontal cortex in schizophrenia Mol Psychiatry 2004 9 299 307 10.1038/sj.mp.4001434 14569272

[B40] Chiba S Hashimoto R Hattori S Yohda M Lipska B Weinberger DR Kunugi H Effect of antipsychotic drugs on DISC1 and dysbindin expression in mouse frontal cortex and hippocampus J Neural Transm 2006 113 1337 1346 10.1007/s00702-005-0414-1 16463116

[B41] Willard HF X chromosome inactivation and X-linked mental retardation Am J Med Genet 1996 64 21 26 10.1002/(SICI)1096-8628(19960712)64:1<21::AID-AJMG2>3.0.CO;2-U 8826443

[B42] Beri S Tonna N Menozzi G Bonaglia MC Sala C Giorda R DNA methylation regulates tissue-specific expression of Shank3 J Neurochem 2007 101 1380 1391 10.1111/j.1471-4159.2007.04539.x 17419801

[B43] Maunakea AK Nagarajan RP Bilenky M Ballinger TJ D'Souza C Fouse SD Johnson BE Hong C Nielsen C Zhao Y Turecki G Delaney A Varhol R Thiessen N Shchors K Heine VM Rowitch DH Xing X Fiore C Schillebeeckx M Jones SJ Haussler D Marra MA Hirst M Wang T Costello JF Conserved role of intragenic DNA methylation in regulating alternative promoters Nature 2010 466 253 257 10.1038/nature09165 20613842 PMC3998662

[B44] Abu-Elneel K Liu T Gazzaniga FS Nishimura Y Wall DP Geschwind DH Lao K Kosik KS Heterogeneous dysregulation of microRNAs across the autism spectrum Neurogenetics 2008 9 153 161 10.1007/s10048-008-0133-5 18563458

